# Reducing the overall use of broad-spectrum antibiotics in NICU is associated with less prevalence of multi-drug resistant *Klebsiella pneumoniae* isolation in premature infants

**DOI:** 10.1186/s13756-025-01693-5

**Published:** 2025-12-28

**Authors:** Meiyan Chu, Jing Lin, Mingjie Wang, Zhengchang Liao, Chuanding Cao, Ying Ding, Yang Liu, Shaojie Yue

**Affiliations:** 1 Department of Neonatology, Xiangya Hospital of Central South University, Xiangya Road 87, Changsha, 410008 Hunan China; 2 Department of Pediatrics, Icahn School of Medicine at Mount Sinai, State of New York, One Gustave L Levy Place, New York, 10029-6574 USA

**Keywords:** Antimicrobial resistance, Klebsiella pneumonia, Preterm infants, Multidrug-resistant

## Abstract

**Objectives:**

The aim of this study was to evaluate the impact of an antibiotic stewardship program on the resistance profiles of *Klebsiella pneumoniae* (*K. pneumoniae*) in the neonatal intensive care unit (NICU). This was achieved by examining changes in the antimicrobial resistance patterns of *K. pneumoniae* isolates collected from preterm infants in a level 3 NICU between 2013 and 2022.

**Methods:**

We examined antibiotic resistance patterns in all isolated *K. pneumoniae* strains cultured from preterm infants (gestational age < 37 weeks, postnatal age > 3 days) admitted to our NICU between 2013 and 2022. To assess temporal trends, we divided the study period into three phases (2013–2015, 2016–2018, and 2019–2022) to correlate antibiotic usage trends with resistance dynamics. Multivariable logistic regression was performed to identify risk factors associated with prevalence of multidrug-resistant (MDR) *K. pneumoniae* colonization or infection in the NICU.

**Results:**

Antimicrobial use, measured in defined daily doses(DDDs) per 100 patient-days, dropped sharply from 30.8 during 2013–2015 to 12.9 in 2016–2018, then fell further to 6.9 in 2019–2022. The rates of MDR *K. pneumoniae* isolation among all isolated *K. pneumoniae* strains decreased significantly from 57.7% in 2013–2015 to 31.8% in 2019–2022 (*p* = 0.001). The resistance rate to aztreonam declined from 60.8% during 2013–2015 to 41.0% in 2016–2018, then further decreased to 25.0% in 2019–2022. The resistance rate to cefepime decreased from 70.1% to 33.3% and finally to 10.2% across the same periods. The resistance rate to piperacillin-tazobactam declined significantly from 53.6% (2013–2015) to 15.2% (2016–2018) and further to 6.8% (2019–2022). The resistance rate to imipenem also declined from 51.5% (2013–2015) to 4.2% (2016–2018) and ultimately to 1.1% (2019–2022). Multivariable logistic regression analysis reveales that exposure to third-generation or higher cephalosporins (aOR = 2.460, 95%CI: 1.386 to 4.365, *p* = 0.002) or glycopeptide antibiotics (aOR = 1.887, 95%CI: 1.100 to 3.237, *p* = 0.021) prior to specimen collection increases the risk of isolating MDR *K. pneumonia* strains.

**Conclusions:**

This study demonstrates that reducing broad-spectrum antibiotic use in NICUs may lower the prevalence of MDR *K. pneumoniae* colonization and infection in premature infants.

## Introduction

 Antimicrobial resistance has become a critical threat to global health, prompting the World Health Organization (WHO) to urge nations to enhance antibiotic resistance surveillance [1]. China confronts particularly serious challenges posed by drug-resistant bacterial infections [2]. Surveillance data from the China Antimicrobial Surveillance Network(CHINET) reveal that multidrug resistance in *Klebsiella pneumoniae* (*K. pneumoniae*) has increased dramatically, with imipenem and meropenem resistance rates rising from 3.0% to 2.9% in 2015 to 22.6% and 23.4% in 2024, respectively. (CHINET, https://www.chinets.com/Data/GermYear).

Implementing antibiotic stewardship programs(ASPs) alongside rigorous infection control measures represents a critical strategy for mitigating the emergence and spread of resistant bacterial strains, since inappropriate antibiotic exposure directly promotes resistance development [3]. However, reducing antibiotic use in intensive care units remains challenging, particularly in neonatal settings, where preterm infants often require invasive interventions such as central venous catheterization, and have a high risk of infection due to their immature immune systems [4]. According to Chinese multicenter data [5] from 2015 to 2018, 88.4% of the preterm infants with gestational age less than 34^0/7^ weeks admitted into tertiary neonatal intensive care units (NICUs) received antibiotic therapy. A meta-analysis by Li et al. [6] of neonatal sepsis studies from Chinese journals (2009–2014) demonstrated that over 50% of Gram-negative isolates, especially *Escherichia* and *Klebsiella* species, exhibited resistance to third-generation cephalosporins.

*K. pneumoniae* remains the predominant pathogen for nosocomial infections in NICUs [7]. It frequently colonizes hospital environments, spreading among patients through contaminated surfaces or medical equipment such as catheters and ventilators [8]. Persistent exposure to antibiotics in intensive care units exerts continuous selective pressure [9], driving the development of diverse genetic resistance mechanisms [10]. Since 2015, our NICU’s antibiotic stewardship program has substantially reduced empirical antibiotic use in the very low birth weight (VLBW) infants, as previously documented [11]. The aim of this study was to evaluate the impact of these interventions on the resistance profiles of *K. pneumoniae* in our NICU by analyzing antimicrobial susceptibility trends in isolates collected from preterm infants between 2013 and 2022.

## Materials and methods

### Study subjects and inclusion criteria

This is a single-center retrospective cohort study performed in the NICU of Xiangya Hospital of Central South University, China. To investigate whether reduced antibiotic usage was associated with a decreased prevalence of multidrug-resistant (MDR) *K. pneumoniae* strains, we analyzed antibiotic resistance patterns of all isolated *K. pneumoniae* strains from all cultures of preterm infants (with gestational age < 37 weeks and postnatal age > 3 days) admitted to the NICU between from January 1, 2013, to December 31, 2022. For patients with multiple cultures yielding identical results, only one specimen per patient was included in the final analysis to eliminate data duplication.

Although the ASP was launched in January 2015, its implementation was fraught with challenges throughout the entire year. Following multiple adjustments to the implementation strategies, the program was not effectively executed until 2016. Consequently, the present study was divided into three phases: 2013–2015, 2016–2018, and 2019–2022. The period from 2016 to 2022 was further split into two phases primarily because the progressive refinement of ASP implementation strategies and the concomitant gradual reduction in antibiotic consumption. This three-phase grouping was designed to enable a staged observation of the dynamic changes in *K.pneumoniae* resistant patterns within the NICU against the context of declining antibiotic use. The specific details of the ASP have been elaborated in a prior publication by our group [12–14]. For MDR *K. pneumoniae* isolates, we performed comparative analyses and logistic regression on clinical parameters and antibiotic exposure histories to identify factors associated with the development of antimicrobial resistance.

The study was approved by the Ethics Committee of Xiangya Hospital of Central South University and was conducted in strict adherence to medical ethics standards. Clinical data were collected retrospectively from the hospital’s medical records. A waiver of parental informed consent was granted by the Institutional Ethics Committee (Ethical Approval No.: 202112258).

### Relevant definitions

Antibiotic use in the NICU was monitored monthly and quantified as defined daily doses per 100 patient-days (DDDs/100PDs). This calculation employed the Anatomical Therapeutic Chemical classification system and WHO-defined daily dose methodology (https://atcddd.fhi.no/atc_ddd_index/). The *K. pneumoniae* isolates collected at our unit were subjected to susceptibility testing against carbapenems (ertapenem, imipenem, and meropenem) and other antimicrobial agents using the bioMerieux VITEK-2 system [15]. Results were interpreted in accordance with the Clinical and Laboratory Standards Institute (CLSI) guidelines [16]. To more accurately assess antimicrobial resistance rates, this study reports both the number of resistant cases and the total number of susceptibility tests performed for each antimicrobial agent, as not all agents were evaluated for every isolate. The resistance rate of *K. pneumoniae* to a given antimicrobial agent was calculated by dividing the number of *K. pneumoniae* strains resistant to the antimicrobial agent during the study period by the total number of isolates tested for that agent, multiplied by 100%. The prevalence of MDR *K. pneumoniae* was calculated as the percentage of MDR isolates among all detected *K. pneumoniae* strains during the study period. MDR *K. pneumoniae* is defined as non-susceptible to at least one agent in three or more antimicrobial categories, as described by Magiorakos et al. [17]. Prolonged rupture of membranes (PROM) is defined as membrane rupture lasting over 18 h. Necrotizing enterocolitis (NEC) was diagnosed according to the revised Bell criteria for stage 2B or higher [18]. Severe bronchopulmonary dysplasia (BPD) was classified according to the NICHD 2018 revised diagnostic criteria [19]. Severe intraventricular hemorrhage (IVH) refers to those with grade III or IV hemorrhages confirmed by cranial ultrasound or brain magnetic resonance imaging [20].

### Statistical analysis

Statistical analyses were conducted with SPSS 22.0 statistical software (SPSS, Inc.,Chicago, IL, USA). Normally distributed data are presented as mean and standard deviation (X ± SD), while non-normally distributed variables are expressed as median and interquartile range (IQR, 25th–75th percentiles). The t-test assessed normally distributed variables in comparisons between the two groups. The Mann-Whitney U test was used for non-normally distributed variables. Categorical data are presented as percentages (%) and compared between groups using the Chi-square test, Fisher’s exact probability test, or Chi-square partitioning method. For multiple comparisons across groups, the significance level was adjusted to *p* < 0.0167. Multivariable logistic regression analysis was used to identify the risk factors for MDR *K. pneumoniae*, correcting for confounding factors such as gestational age, birth weight, Apgar scores, etc. A two-tailed *p* < 0.05 was considered statistical significant.

## Results

### Decreased DDDs of antibiotics in the NICU during the study periods

**Fig. 1:**
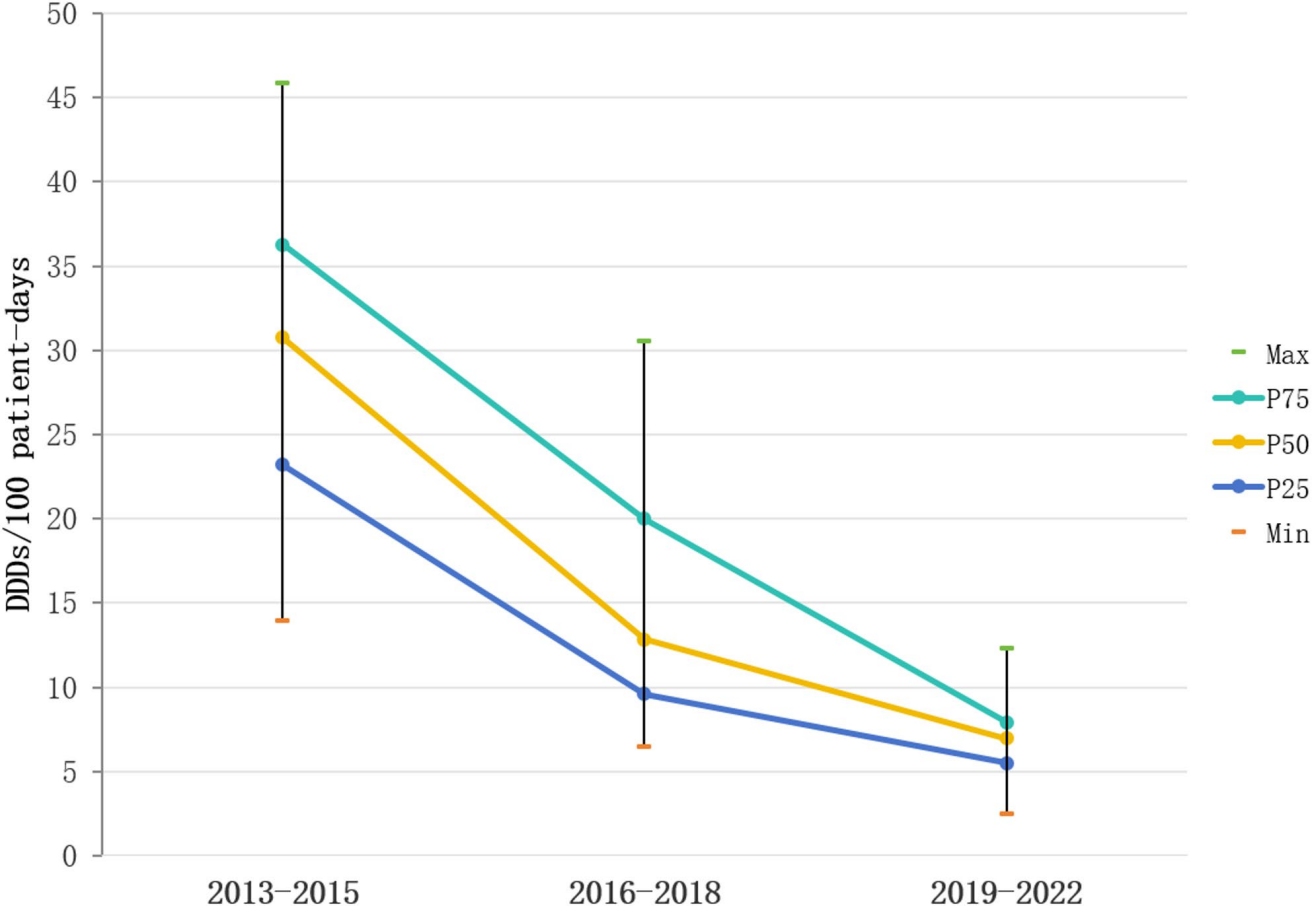
DDDs of antibiotics in the NICU across three distinct periods

The NICU at Xiangya Hospital of Central South University implemented a rigorous antibiotic stewardship program, which progressively reduced antimicrobial exposure among premature infants [11]. To quantify these changes, we analyzed the antibiotic consumption data from 2013 to 2022 using defined daily doses (DDDs). Figure 1 shows a significant reduction in overall antibiotic use, with median DDDs values decreased from 30.8 during 2013–2015 to 12.9 in 2016–2018, and further down to 6.9 per 100 patient-days in 2019–2022 (*p* < 0.001). These results confirm sustained reductions in antibiotic utilization in our NICU throughout the study period.

### Significant reduction in antibiotic resistance of *K. pneumoniae* in the NICU

To assess the impact of reduced antibiotic use on *K. pneumoniae* resistance in the NICU, we collected and analyzed all *K. pneumoniae*-positive cultures (either from infection or colonization) isolated from preterm infants (gestational age < 37 weeks and postnatal age > 3 days) between 2013 and 2022. For patients with multiple cultures yielding identical *K. pneumoniae* cultures, only one specimen per patient was included to avoid duplication. A total of 329 *K. pneumoniae* strains were cultured during the study period. Most isolates were obtained from respiratory secretions, likely representing colonization, while 121 (36.8%) were derived from blood or other sterile body fluids, indicating true infections. Table 1 demonstrates a marked decrease in the prevalence of MDR *K. pneumoniae* across the three study periods (2013–2015, 2016–2018, and 2019–2022), with the resistance rate decreased from 57.7% to 31.8% (*p* = 0.001).

Temporal declines in the antibiotic resistance patterns of isolated *K. pneumoniae* were significant. The resistance rate to aztreonam decreased from 60.8% during 2013–2015 to 41.0% in 2016–2018, then further decreased to 25.0% in 2019–2022 (*p* < 0.001). Resistance to ceftriaxone decreased from 80.4% (2013–2015) to 54.9% (2016–2018) and subsequently to 39.8% (2019–2022) (*p* < 0.001). Similarly, the resistance rate to ceftazidime decreased from 65.0% (2013–2015) to 29.7% (2019–2022) (*p* < 0.001). The most significant reduction was observed in resistance to cefepime, with rates falling from 70.1% (2013–2015) to 33.3% (2016–2018) and finally to 10.2% (2019–2022) (*p* < 0.001) (Table 1).

The resistance rate of *K. pneumoniae* to piperacillin-tazobactam declined sharply from 53.6% (2013–2015) to 15.2% (2016–2018) and further to 6.8% (2019–2022) (*p* < 0.001). Resistance to ampicillin-sulbactam decreased from 78.8% (2013–2015) to 56.8% (2019–2022) (*p* = 0.005). Resistance to cefoperazone-sulbactam exhibited a similar downward trend, declining from 61.1% (2013–2015) to 11.6% (2016–2018) and subsequently to 4.6% (2019–2022) (*p* < 0.001). The resistance rate to imipenem-a key carbapenem antibiotic-also showed a pronounced decline, falling from 51.5% (2013–2015) to 4.2% (2016–2018) and ultimately to 1.1% (2019–2022) (*p* < 0.001) (Table 1).

Despite the significant decrease in the resistance rates of *K. pneumoniae* to commonly prescribed antibiotics, the proportion of MDR *K. pneumoniae* isolates remained relatively high at levels > 30% in 2019–2022.

**Table 1:** Comparison of antibiotic resistance of *K. pneumoniae* among the three distinct periods

Antimicrobal agent	2013–2015(*n* = 97)	2016–2018(*n* = 144)	2019–2022(*n* = 88)		*p*
Multidrug resistance	56/97(57.7)	58/144(40.3) ^*^	28/88(31.8) ^*^	*χ*^*2*^ = 13.498	0.001
Aztreonam	59/97(60.8)	59/144(41.0) ^*^	22/88(25.0) ^*^	*χ*^*2*^ = 24.486	<0.001
Amikacin	0/97(0.0)	0/144 (0.0)	0/88 (0.0)		1.000
Ciprofloxacin	2/97(2.1)	1/143(0.7)	24/88(27.3) ^*#^	*χ*^*2*^ = 57.864	<0.001
Ceftriaxone	78/97(80.4)	79/144(54.9) ^*^	35/88(39.8) ^*#^	*χ*^2^ = 32.647	<0.001
Cefepime	68/97(70.1)	48/144(33.3) ^*^	9/88(10.2) ^*#^	*χ*^*2*^ = 72.577	<0.001
Ceftazidime	52/80(65.0)	57/141(40.4) ^*^	19/64(29.7) ^*^	*χ*^*2*^ = 20.191	<0.001
Cefazolin	73/90(81.1)	88/141(62.4) ^*^	54/88(61.4) ^*^	*χ*^*2*^ = 10.756	0.005
Ampicillin	97/97(100.0)	144/144(100.0)	88/88(100.0)		1.000
Piperacillin/Tazobactam	52/97(53.6)	20/132(15.2) ^*^	6/88(6.8) ^*^	*χ*^*2*^ = 65.350	<0.001
Ampicillin/Sulbactam	63/80(78.8)	95/130(73.1)	50/88(56.8) ^*#^	*χ*^*2*^ = 10.737	0.005
Cefoperazone/Sulbactam	58/95(61.1)	16/138(11.6) ^*^	4/87(4.6) ^*^	*χ*^*2*^ = 100.019	<0.001
Imipenem	50/97(51.5)	6/144(4.2) ^*^	1/88(1.1) ^*^	*χ*^*2*^ = 112.815	<0.001
Gentamicin	28/96(29.2)	7/144(4.9) ^*^	11/88(12.5) ^*^	*χ*^*2*^ = 28.453	<0.001
Tobramycin	6/96(6.3)	2/134(1.5)	0/88(0.0) ^*^	*χ*^*2*^ = 6.884	0.022
Levofloxacin	1/97(1.0)	1/144(0.7)	6/88(6.8) ^*#^	*χ*^*2*^ = 7.627	0.013
Bactrim	29/97(29.9)	59/144(41.0)	34/88(38.6)	*χ*^*2*^ = 3.172	0.205
Nitrofurantoin	36/96(37.5)	46/144(31.9)	19/53(35.8)	*χ*^*2*^ = 0.841	0.630

^*^: compared with 2013–2015 group: adjusted *p*<0.05; ^#^: compared with 2016–2018: adjusted *p*<0.05

### Factors associated with multi-drug resistance in *K. pneumoniae* isolates from the NICU

To investigate factors associated with the isolation of MDR *K. pneumoniae* strains among all cases, we analyzed clinical data from all cases with confirmed *K. pneumoniae* infection or colonization (Table 2). Gestational age, birth weight, sex, Apgar score, central venous catheterization, ventilator use, mortality and others clinical variables showed no significant differences between infants with cultured MDR strains and those with non-MDR strains. However, NEC occurred more frequently in the group with non-MDR *K. pneumoniae* isolation (28.3% vs. 16.9%, *p* = 0.015).

**Table 2:** Clinical data of preterm infants with cultured non-MDR and MDR *K. pneumoniae* strains

Variables	Non-MDR(*n* = 187)	MDR(*n* = 142)		*p*
Neonatal characteristics				
GA^①^, mean (SD) (w)	29.3 ± 2.6	29.6 ± 2.5	*t* = 1.281	0.201
BW^②^, mean (SD) (g)	1342.2 ± 497.3	1344.3 ± 535.5	*t* = 0.038	0.970
Male, *n* (%)	109(58.3)	90(63.4)	*χ*^*2*^ = 0.875	0.349
IVF^③^, *n* (%)	68(36.6)	50(35.2)	*χ*^*2*^ = 0.064	0.801
Apgar score ≤ 7 at 1 min	8.0(5.0, 9.0)	8.0(5.0, 9.0)	*Z* = 1.447	0.148
Apgar score ≤ 7 at 5 min	9.0(8.0, 10.0)	9.0(8.0, 10.0)	*Z* = 0.134	0.894
Apgar score ≤ 7 at 10 min	10.0(9.0, 10.0)	10.0(9.0, 10.0)	*Z* = 1.819	0.069
Central venous catheterization, *n* (%)	153(82.3)	122(86.5)	*χ*^*2*^ = 1.092	0.296
Surfactant usage, *n* (%)	143(77.3)	109(81.3)	*χ*^*2*^ = 0.767	0.381
Non-invasive ventilation ≥ 7 days before specimen collection, *n* (%)	133(71.1)	102(72.3)	*χ*^*2*^ = 0.059	0.809
Invasive ventilation ≥ 7 days before specimen collection, *n* (%)	24(12.8)	21(15.2)	*χ*^*2*^ = 0.378	0.539
Age of the specimen was collected(d)	26.0(12.0, 40.0)	18.5(11.0, 38.3)	*Z* = 1.087	0.227
Number of days of central venous use(d)	20.0(9.0, 28.5)	15.0(6.0, 29.0)	*Z* = 0.986	0.324
Number of days of non-invasive ventilation before specimen collection(d)	13.0(6.0, 27.0)	11.0(5.0, 25.0)	*Z* = 1.119	0.263
Number of days of invasive ventilation before specimen collection(d)	0.0(0.0, 2.0)	0.0(0.0, 2.0)	*Z* = 1.267	0.205
Death, *n* (%)	26(13.9)	16(11.3)	*χ*^*2*^ = 0.504	0.478
Moderate to Severe BPD^④^, *n* (%)	35(21.2)	32(24.6)	*χ*^*2*^ = 0.480	0.489
≥ 2B NEC^⑤^, *n* (%)	53(28.3)	24(16.9)	*χ*^*2*^ = 5.893	0.015
Severe IVH^⑥^, *n* (%)	17(9.1)	16(11.3)	*χ*^*2*^ = 0.424	0.515

**Maternal characteristics**				
Vaginal delivery, *n* (%)	65(34.8)	48(33.8)	*χ*^*2*^ = 0.033	0.856
PROM^⑦^, *n* (%)	58(31.0)	46(32.4)	*χ*^*2*^ = 0.071	0.790
Pre-eclampsia, *n* (%)	25(13.4)	28(19.7)	*χ*^*2*^ = 2.408	0.121
Gestational diabetes, *n* (%)	10(5.3)	8(5.6)	*χ*^*2*^ = 0.013	1.000

^①^: Gestational age; ^②^: Birth weight; ^③^: In vitro fertilization; ^④^: Bronchopulmonary dysplasia; ^⑤^: Necrotizing enterocolitis; ^⑥^: Intraventricular hemorrhage; ^⑦^: Prolonged rupture of membranes

There was no significant difference in antibiotic usage prior to specimen collection between the two groups (93.0% vs. 95.8%, *p* = 0.294). However, the non-MDR *K. pneumoniae* group showed significantly lower utilization rates than the MDR *K. pneumoniae* group for both third-generation or higher cephalosporins (16.0% vs. 31.7%, *p* = 0.001) and glycopeptide antibiotics (26.2% vs. 40.8%, *p* = 0.005) (Table 3).

**Table 3:** Antibiotic use prior to specimen collection in preterm infants with cultured non-MDR and MDR *K. pneumoniae* strains

Antibiotic Use	Non-MDR(*n* = 187)	MDR(*n* = 142)		*p*
Antibiotic Use prior to specimen collection, *n* (%)	174(93.0)	136(95.8)	*χ*^*2*^ = 1.103	0.294
β-Lactamaseinhibitors, *n* (%)	143(76.5)	120(84.5)	*χ*^*2*^ = 3.251	0.071
Carbapenem, *n* (%)	74(39.6)	70(49.3)	*χ*^*2*^ = 3.101	0.078
Third-generation or higher cephalosporins, *n* (%)	30(16.0)	45(31.7)	*χ*^*2*^ = 11.228	0.001
Glycopeptide, *n* (%)	49(26.2)	58(40.8)	*χ*^*2*^ = 7.885	0.005

To investigate whether antibiotic exposure prior to specimen collection increases the risk of isolating MDR *K. pneumoniae*, we conducted a multivariate logistic regression analysis adjusted for gestational age, birth weight, Apgar score at 1 min, and other confounding variables. Exposure to third-generation or higher cephalosporins (aOR = 2.460, 95%CI: 1.386 to 4.365, *p* = 0.002) or glycopeptide antibiotics (aOR = 1.887, 95%CI: 1.100 to 3.237, *p* = 0.021) prior to specimen collection was associated with an increased odds of isolating an MDR *K. pneumoniae* strain. Compared with the 2013–2015 period, the adjusted odds ratio declined significantly in 2016–2018 (aOR = 0.438, 95%CI: 0.243 to 0.790, *p* = 0.006) and 2019–2022 (aOR = 0.264, 95%CI: 0.132 to 0.527, *p* < 0.001), a trend likely attributable to reduced overall antibiotic utilization in the NICU (Table 4).

**Table 4:** Logistic regression analysis of risk factors for isolating MDR *K. pneumonia* in preterm infants

Variables	ALL(*n* = 329)		aOR (95%CI)	*p*
	Non-MDR(*n* = 187)	MDR(*n* = 142)		
**Antimicrobial use before specimen collection**				
β-Lactamaseinhibitors	143(76.5)	120(84.5)	1.709(0.912, 3.201)	0.094
Carbapenem	74(39.6)	70(49.3)	1.453(0.874, 2.416)	0.150
Third-generation or higher cephalosporins	30(16.0)	45(31.7)	2.460(1.386, 4.365)	0.002
Glycopeptide	49(26.2)	58(40.8)	1.887(1.100, 3.237)	0.021
**Years**				
2013 - 2015	41(21.9)	56(39.4)	1	
2016 - 2018	86(46.0)	58(40.8)	0.438(0.243, 0.790)	0.006
2019 - 2022	60(32.1)	28(19.7)	0.264(0.132, 0.527)	<0.001

Adjusted factors: gestational age at birth, birth weight (Kg), Apgar score at 1 min, male, vaginal delivery, IVF, premature rupture of membranes, central venous catheterization before specimen collection, pulmonary surfactant, usage of non-invasive ventilator ≥ 7 days before specimen collection, and usage of invasive ventilator ≥ 7 days before specimen collection

## Discussion

Neonatal bacterial infections present diagnostic difficulties due to overlapping symptoms with non-infectious conditions, particularly in premature and immunocompromised infants. The nonspecific clinical presentation frequently delays accurate diagnosis, whereas untreated sepsis carries a substantial risk of mortality. These challenges have contributed to the widespread use of empirical antibiotics and overprescription of broad-spectrum antimicrobial agents in NICUs. It has been demonstrated that only a small proportion of antibiotic administration in NICUs is targeted at confirmed infection, highlighting the urgent need for ASPs [21]. Starting in 2015, we have initiated and implemented an ASP in the NICU, which resulted in a significant reduction in antibiotic use among VLBW infants in 2016 [11]. Our current study found that antibiotic use in the NICU, quantified by DDDs, decreased consistently over the study period, thus validating the efficacy of ASPs. Associated with the reduction in overall antibiotic utilization in the NICU, the proportion of MDR *K. pneumoniae* isolates also decreased significantly during this period. Specifically, the proportion of MDR *K. pneumoniae* isolates dropped from 57.7% during 2013–2015 to 31.8% in 2019–2022 (*p* = 0.001), demonstrating a clear temporal trend toward reduced antimicrobial resistance. Multivariate logistic regression analysis indicates that reduced antibiotic use correlates with a lower proportion of MDR *K. pneumoniae*. Furthermore, exposure to broad-spectrum antibiotics prior to specimen collection may be associated with an increased likelihood of isolating a strain of MDR *K. pneumoniae*.

As the leading pathogen responsible for nosocomial infections in NICUs, *K. pneumoniae* can cause severe infections such as late onset sepsis, ventilator associated pneumonia, urinary tract infections, and meningitis in neonates—especially premature or immunocompromised infants. The rise of antimicrobial resistance, particularly to extended-spectrum beta-lactams (ESBLs) and carbapenems, poses formidable challenges to clinical treatment and infection control practices [22, 23]. MDR *K. pneumoniae* exhibits resistance to multiple classes of antibiotics, primarily through enzymatic degradation, altered membrane permeability, target site modifications, and efflux pump mechanisms [24]. These resistance traits are often plasmid-mediated, facilitating horizontal gene transfer and rapid dissemination—especially in intensive care units settings [25]. Selective pressure from antibiotic abuse drives the emergence of multidrug resistance in *K. pneumoniae* [26]. The escalation of MDR bacterial colonization and infections in neonates, driven by antibiotic overuse, not only complicates treatment but also imposes substantial economic burdens, particularly in developing countries [27].

It is noteworthy that the problem of drug resistance in *K. pneumoniae* in NICUs remains severe. Clinical isolates from three Ethiopian tertiary hospitals [28] demonstrated ESBL production in 92.1% of cases and carbapenemase production in 47.4%, with resistance rates ranging from 91% to 100% for cephalosporins, 49.1% for meropenem, and 21% for amikacin. Although the proportion of MDR *K. pneumoniae* in our NICU declined markedly during the study period, with reduced resistance rates to aztreonam, third- or higher-generation of cephalosporins, piperacillin-tazobactam, and carbapenems, over 30% of recent isolates remained MDR. Notably, resistance persisted to cefazolin and ampicillin-sulbactam, two key first-line antibiotics indicated for neonatal treatment. The high prevalence of antibiotic resistance poses a serious clinical challenge, which limits therapeutic options and increases mortality risks in affected neonates.

In response to the current dire situation of growing threat of antibiotic resistance, implementing ASPs in clinical practice remains the most viable approach pending the development of new effective antibiotics. While clinical studies have demonstrated that antibiotic reduction strategies effectively curb the emergence and transmission of resistant bacteria, the implementation of ASPs in NICUs continues to face practical challenges. A retrospective quasi-experimental study [29] conducted at An-Najah National University Hospital, a tertiary care center in the West Bank, Palestine, assessed the efficacy of ASPs. The results demonstrate statistically significant improvements in Pseudomonas aeruginosa susceptibility to meropenem, piperacillin, and piperacillin-tazobactam following ASP implementation. Sean et al. [30] employed agent-based modeling to simulate patient-healthcare worker interactions, showing that reducing antibiotic use by 10% (from 75% to 65%) and 25% (from 75% to 50%) lowered the acquisition rates of prevalent MDR organisms by 11.2% (*p* < 0.001) and 28.3% (*p* < 0.001), respectively. The slow development of novel antibiotics—particularly those safe for neonatal use—emphasizes the critical need for stringent infection control measures and the minimization of broad-spectrum antibiotic overuse in NICUs via ASPs. The findings of this study demonstrate that reducing unnecessary antibiotic use is feasible and can significantly suppress the emergence of multidrug resistance in *K. pneumoniae*—one of the most common nosocomial pathogens in NICUs. This highlights the imperative for clinicians to exercise caution, especially when prescribing broad-spectrum antibiotics.

This retrospective study has limitations. First, the DDDs of antibiotics were provided by the medical administration department of our hospital. Notably, the calculation of DDDs does not adopt a pediatric-specific standard; additionally, the required antibiotic doses for neonates are relatively low. Therefore, the DDDs in this study cannot be directly compared with those from adult departments or other NICUs. Second, the *K. pneumoniae* isolates in this study were derived from a wide range of clinical specimens, including blood, cerebrospinal fluid, and sputum. Among these isolates, the majority of *K. pneumoniae* strains were identified as colonizing bacteria. Given that the primary objective of this research was to investigate the impact of reduced antibiotic consumption on the antibiotic resistance rate of *K. pneumoniae* in the NICU, we did not distinguish between colonizing and infecting strains. We therefore advise readers to avoid overinterpretation of the study findings. It is our hope that this study will encourage NICU clinicians to exercise greater prudence in antibiotic prescribing—particularly for broad-spectrum agents—and to acknowledge the potential benefits of implementing ASPs.

## Conclusions

With the implementation of an ASP in the NICU, overall antibiotic consumption quantified by DDDs showed a significant decline from 2013 to 2022. Concomitant with the reduction in DDDs, the resistance rates of *K. pneumoniae* to aztreonam, ceftriaxone, ceftazidime, cefepime, piperacillin-tazobactam, ampicillin-sulbactam, cefoperazone-sulbactam, and imipenem all decreased significantly. The proportion of MDR strains among all *K. pneumoniae* isolates declined significantly in 2016–2018 and 2019–2022 compared with the 2013–2015 period. Multivariate logistic regression analysis indicates that exposure to third- or higher-generation cephalosporins or glycopeptide antibiotics prior to specimen collection increases the risk of isolating an MDR *K. pneumoniae* strain. This study demonstrates that restricting broad-spectrum antibiotic use in NICUs may decrease prevalence of MDR *K. pneumoniae* colonization or infection in this setting.

## Data Availability

The data are available on request.

## Abbreviations

ASP: Antibiotic stewardship programs
BW: Birth weight
BPD: Bronchopulmonary dysplasia
DDDs: Defined daily doses
GA: Gestational age
IVH: Intraventricular hemorrhage
IVF: In vitro fertilization
MDR: Multidrug-resistant
NEC: Necrotizing enterocolitis
NICU: Neonatal intensive care unit
PROM: Prolonged rupture of membranes
VLBW: Very low birth weight
